# Proteomic Profiling of Endothelial Cells Exposed to Mitomycin C: Key Proteins and Pathways Underlying Genotoxic Stress-Induced Endothelial Dysfunction

**DOI:** 10.3390/ijms25074044

**Published:** 2024-04-05

**Authors:** Maxim Sinitsky, Egor Repkin, Anna Sinitskaya, Victoria Markova, Daria Shishkova, Olga Barbarash

**Affiliations:** 1 Laboratory of Genome Medicine, Research Institute for Complex Issues of Cardiovascular Diseases, 6 Academician Barbarash Boulevard, 650002 Kemerovo, Russia; 2 Centre for Molecular and Cell Technologies, St. Petersburg State University, 7/9 Universitetskaya Embankment, 199034 St. Petersburg, Russia; 3 Laboratory for Molecular, Translation and Digital Medicine, Research Institute for Complex Issues of Cardiovascular Diseases, 6 Academician Barbarash Boulevard, 650002 Kemerovo, Russia; 4 Research Institute for Complex Issues of Cardiovascular Diseases, 6 Academician Barbarash Boulevard, 650002 Kemerovo, Russia

**Keywords:** mutagenesis, atherogenesis, endothelial disfunction, genotoxic stress, DNA damage, proteome, mass spectrometry, bioinformatic analysis, differentially expressed proteins

## Abstract

Mitomycin C (MMC)-induced genotoxic stress can be considered to be a novel trigger of endothelial dysfunction and atherosclerosis—a leading cause of cardiovascular morbidity and mortality worldwide. Given the increasing genotoxic load on the human organism, the decryption of the molecular pathways underlying genotoxic stress-induced endothelial dysfunction could improve our understanding of the role of genotoxic stress in atherogenesis. Here, we performed a proteomic profiling of human coronary artery endothelial cells (HCAECs) and human internal thoracic endothelial cells (HITAECs) in vitro that were exposed to MMC to identify the biochemical pathways and proteins underlying genotoxic stress-induced endothelial dysfunction. We denoted 198 and 71 unique, differentially expressed proteins (DEPs) in the MMC-treated HCAECs and HITAECs, respectively; only 4 DEPs were identified in both the HCAECs and HITAECs. In the MMC-treated HCAECs, 44.5% of the DEPs were upregulated and 55.5% of the DEPs were downregulated, while in HITAECs, these percentages were 72% and 28%, respectively. The denoted DEPs are involved in the processes of nucleotides and RNA metabolism, vesicle-mediated transport, post-translation protein modification, cell cycle control, the transport of small molecules, transcription and signal transduction. The obtained results could improve our understanding of the fundamental basis of atherogenesis and help in the justification of genotoxic stress as a risk factor for atherosclerosis.

## 1. Introduction

Genotoxic stress in mammalian cells is defined as a situation that initiates DNA damage compromising the cell’s genomic integrity leading to replication and transcription arrest [1] and underlies many pathological conditions including cellular senescence [2,3,4,5], cancer [6,7,8,9] and cardiovascular diseases [10,11,12,13,14]. Recent experimental data suggest that the genotoxic stress in vitro induced by alkylating mutagen mitomycin C (MMC) is associated with the proinflammatory activation of primary human endothelial cells and the endothelial-to-mesenchymal transition [15,16,17], the key pathways underlying endothelial disfunction [18]—an initial stage of atherosclerosis [19,20], a leading cause of cardiovascular morbidity and mortality worldwide [21,22]. This finding makes it possible to consider genotoxic stress as a novel risk factor for atherosclerosis, but it is still not completely known what molecular mechanisms, processes and signaling pathways underlie the proatherosclerotic transformation of endothelial cells in response to genotoxic stress.

Given the increasing genotoxic load on the human organism from various environmental (ionizing and UV radiation) and anthropogenic (tobacco smoke, exhaust gases, industrial waste) sources [23,24,25,26], the decryption of the molecular mechanisms underlying genotoxic stress-induced endothelial dysfunction has both fundamental and applied significance—it could improve our understanding of atherogenesis, help in the justification of genotoxic stress as a novel risk factor for atherosclerosis and make it possible to develop an effective atherosclerosis therapy targeting the key pathogenetically significant molecular pathways found mainly in populations of regions with high genotoxic backgrounds.

Whole-transcriptome sequencing (RNA-seq) and ultra-high performance liquid chromatography–mass spectrometry (UHPLC-MS/MS) are the modern high-throughput methods allowing for the identification of any signaling pathways determining the acquisition of certain phenotypes by cells in response to various stimuli. Resulting from RNA-seq, MMC-induced genotoxic stress in primary human coronary artery endothelial cells (HCAEC) and internal thoracic artery endothelial cells (HITAEC) leads to the upregulation of genes involved in the p53, GAS6/AXL, JNK/SAPK, PI3K/AKT, DNA damage, oxidative stress and inflammatory response signaling pathways, the inflammatory activation of endothelial cells, endothelial migration and differentiation, the adhesion of mononuclear blood fractions to the plasma membrane of endothelial cells and apoptosis, and to the downregulation of genes involved in angiogenesis [27]. At the same time, the proteomic profiling of the endothelial cells incubated under a genotoxic load has still not been performed.

The presented research is aimed to label-free proteomic profiling of HCAECs and HITAECs in vitro that were exposed to MMC followed by bioinformatic analysis to identify the biochemical pathways and functional proteins underlying genotoxic stress-induced endothelial dysfunction.

## 2. Results

**Figure 1:**
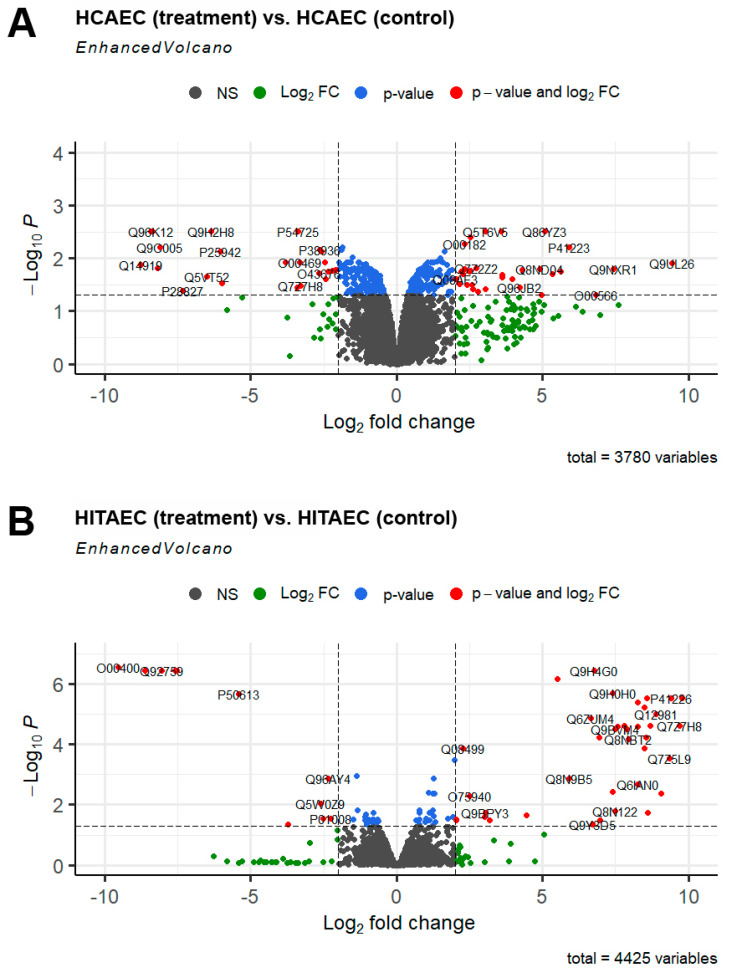
Volcano plot showing the distribution of proteins in the proteome of HCAECs (**A**) and HITAECs (**B**). Gray points—the proteins with a log_2_ fold change < 1 and an FDR-corrected *p*-value > 0.05; green points—the proteins with a log_2_ fold change > 1 and an FDR-corrected *p*-value > 0.05; blue points—the proteins with a log_2_ fold change < 1 and an FDR-corrected *p*-value < 0.05; red points—the proteins with a log_2_ fold change > 1 and an FDR-corrected *p*-value < 0.05 (DEPs).

**Figure 2:**
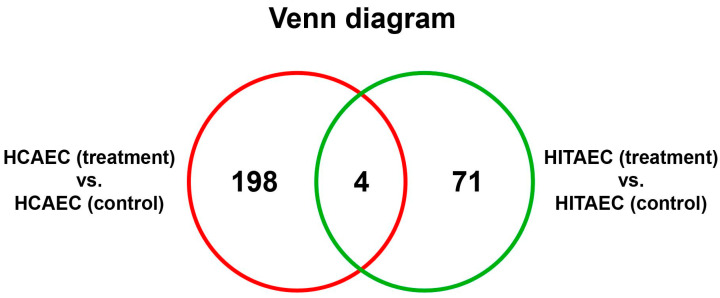
**A** Venn diagram demonstrating the number of unique and common DEPs in the MMC-treated HCAECs and HITAECs.

Resulting from UHPLC-MS/MS followed by bioinformatical analysis, 202 and 75 differentially expressed proteins (DEPs) after applying cut-off criteria (logarithmic fold change > 1 and FDR-corrected *p*-value < 0.05) were denoted in the MMC-treated HCAECs and HITAECs, respectively, compared to the non-exposed control (Figure 1). It should be noted that only four DEPs (RM10, KTAP2, TMED5 and SLFN5) were identified in both the HCAECs and HITAECs (Figure 2). In the MMC-treated HCAECs, 44.5% of the DEPs were upregulated and 55.5% of the DEPs were downregulated, while in the HITAECs, these percentages were 72% and 28%, respectively. A total of 7 DEPs in the HCAECs (RB22A, NDE1, MMP10, BUD31, SYNE1, RAP2C and HORN) and 32 DEPs in the HITAECs (ALBU, T4S1, RABE1, RM10, UBA7, I2BP2, TMED5, SEC20, RL26L, NCOA7, SPAST, CH033, VLDLR, INT7, BPHL, RMC1, DRS7B, SPC24, KTAP2, RIN1, ZMYD8, RPTOR, GGACT, INT2, RPAP3, NAKD2, CAST2, E41L1, RT18C, RHG27, JMY and RN114) were upregulated in a logarithmic fold change >5; 10 DEPs in the HCAECs (COMD2, TNR5, PPIL3, RPRD2, PTPRM, DPY30, KTAP2, OXSM, FACR2 and NC2A) and 7 DEPs in the HITAECs (CDK7, ABCB7, INT14, TF2H4, ATM, ACATN and JAGN1) were downregulated at the same level.

According to Reactome Pathway Database, the denoted DEPs were classified into the 13 most significant (FDR-corrected *p*-value ≤ 0.05) biochemical pathways. In HCAECs, the upregulated DEPs are involved in the processes of nucleotides and RNA metabolism, while the downregulated DEPs are involved in the processes of vesicle-mediated transport, post-translation protein modification and cell cycle control (Table 1). The DEPs downregulated in the MMC-treated HITAECs belong to pathways involved in the transport of small molecules, transcription and signal transduction. The upregulated DEPs in the HITAECs were not classified into any pathways (Table 2).

The identified DEPs were also processed using Gene Ontology (GO) enrichment analysis. GO is a controlled vocabulary containing more than 38,000 precisely defined phrases called GO terms and describing the molecular functions of gene products (molecular functions, MFs), the biological processes in which those functions are involved (biological processes, BPs) and their cellular locations (cellular components, CCs) [28]. According to the GO analysis, the upregulated and downregulated DEPs in the HCAECs were classified into 10 and 8 significant (FDR-corrected *p*-value ≤ 0.05) molecular terms, respectively (Table 3). In the HTAECs, the downregulated DEPs were classified into only one BP group—the positive regulation of the heme biosynthetic process (fold enrichment > 100, FDR-corrected *p*-value = 2.05 × 10^−2^); the upregulated DEPs were not classified into any pathways.

## 3. Discussion

Atherosclerosis, a chronic multifactorial inflammatory pathology of large and medium-sized arteries, is a leading cause of cardiovascular morbidity and mortality worldwide [22]. The pathophysiological basis of this disease is an accumulation of modified lipids, immune cells and cell debris in the subendothelial space of different arteries leading to atherosclerotic plaque formation [29,30]. The forming of a healthy endothelium by the single layer of endothelial cells located in the intima layer of arteries acts as a barrier that separates vessels’ walls from the intravascular flow, regulating vascular tone, inflammatory response and angiogenesis, preventing platelet aggregation, and maintaining fluid homeostasis [31,32,33]. The failure of the endothelium to fully perform these functions is defined as endothelial dysfunction [20]. It is known that endothelial homeostasis can be impaired by a number of triggers, including generally accepted ones such as low or non-laminar shear stress, metabolic and chemical stress [19], as well as genotoxic stress, which has been described recently as a novel risk factor for endothelial dysfunction [15,16,17]. Despite the available experimental data, genotoxic stress is currently not justified as a significant risk factor for endothelial dysfunction and is not considered among the current clinical recommendations for atherosclerosis therapy [34]. Moreover, the molecular mechanisms underlying genotoxic stress-induced endothelial dysfunction are not completely deciphered. Thus, the studying of the pathways involved in the impairing of endothelial homeostasis and the acquisition of the proatherosclerotic phenotype by endothelial cells is important not only for obtaining an understanding of the endothelium’s pathophysiology, but also for the pathogenetic justification of genotoxic stress as a risk factor for endothelial dysfunction and atherosclerosis.

Proteins are the key molecules involved in the catalyzing of a chemical reaction, cell signaling and signal transduction in a human organism [35]. The proteome, defined as the entire set of proteins expressing in cells, tissues or an organism at a certain time, can be analyzed using the proteomics approach that allows us to study any biological processes and pathways in a more detailed manner. The key technology utilized for protein identification in biological samples is UHPLC-MS/MS [36]. Here, we first performed the proteomic profiling and identification of proteins expressed in HCAECs and HITAECs in response to the genotoxic stress induced by MMC.

MMC is a chemotherapy and anti-fibrotic drug characterized by clastogenic activity [37,38,39,40,41,42,43,44]. The ability of MMC to induce genotoxic stress is due to the peculiarities of its metabolism. In mammalian cells, MMC metabolizes into mitosene [45], which reacts with 7-N-guanine nucleotide residues in the minor groove of DNA via N-alkylation resulting in DNA crosslinking [46], replication and transcription arresting, and finally, apoptosis [47].

Resulting from our experiment, the MMC-induced genotoxic stress in HCAECs is associated with the upregulation of proteins involved in nucleotides and RNA metabolism. So, the phosphate bond hydrolysis by NUDT proteins, a member of larger purine/nucleotide catabolism pathway, is the most significantly upregulated pathway. Enzymes that belong to the Nudix hydrolase superfamily can prevent the base mispairing during DNA replication induced by transversions (spontaneous [48] or caused by alkylating agents [49]) via catalyzing the hydrolysis of nucleoside tri- and diphosphates and nucleotide sugars [50,51]. Due to these functions, NUDT proteins may protect the cells from clastogenic damage (if modified deoxyribonucleotides were incorporated into DNA) and from the synthesis of aberrant proteins (if modified ribonucleotides were incorporated into mRNA) [52,53]. Thus, the upregulation of the phosphate bond hydrolysis by NUDT proteins pathway may be a cellular response to MMC treatment to prevent genotoxic stress in endothelial cells.

Another upregulated pathway identified in HCAECs is the binding and destabilizing of mRNA by tristetraprolin (TTP, ZFP36). TTP binds RNA containing AU-rich elements and recruits enzymes that promote RNA degradation, mainly in the following mRNA encoding proinflammatory mediators: *TNFα* (tumor necrosis factor alpha), *CSF2* (granulocyte-macrophage colony stimulating factor), *IL2* (interleukin 2) and FOS (proto-oncogene C-FOS) [54,55,56,57]. It has been shown that mice deficient in TTP exhibit arthritis, weight loss, skin lesions, autoimmunity, and myeloid hyperplasia [58]. Currently, TTP is considered to be a crucial post-transcriptional regulator of inflammation [59]. Considering that MMC-induced genotoxic stress is associated with the proinflammatory activation of endothelial cells [17], the upregulation of the destabilizing of mRNA by TTP pathway in MMC-treated HCAECs may be a compensatory cellular response of endothelial cells to MMC-mediated inflammation. Generally, we can suggest that the upregulation of molecular pathways in HCAECs in response to MMC-induced genotoxic stress is a compensatory mechanism and serves to stabilize endothelial homeostasis and prevent more serious impairments of endothelial cell function leading to their proatherosclerotic transformation. The sealing of the nuclear envelope by ESCRT-III (endosomal sorting complexes required for transport III) is one of the downregulated pathways in the MMC-treated HCAECs. In eukaryotic cells, the nuclear envelope is transiently dissolved followed by the forming of nuclear pore complexes (NPCs) during mitosis and interphase in migrating mammalian cells. The NPCs are allowing nuclear proteins to leak out and cytoplasmic proteins to leak in [60,61]. The formation of NPCs is caused by nuclear deformation and is repaired in an ESCRT-dependent manner [62]. In other words, the ESCRT-III complex is required for the fast resealing of the nucleocytoplasmic barrier [63,64]. It has been shown that diffusing cytoplasmic factors might enter into the nucleus through NPCs and induce DNA damage [65,66,67,68]. Resulting from these findings, it has been suggested that prolonged nuclear pore opening can lead to cell death through DNA damage-induced apoptosis, provided that DNA repair is also affected [61].

The post-translational modification of proteins is very important for activity, the localization and stability of proteins, and protein–protein interactions. The ubiquitination affecting the majority of cellular functions is one of the key methods of the post-translational modification of proteins. Five gene families have been described that encode the enzymes controlling ubiquitination (named the deubiquitinating enzymes or DUBs) by hydrolyzing the isopeptide bond tethering ubiquitin to itself or the target protein resulting in the cleaving of ubiquitin from the substrate [69,70]. The JAB1/MPN+/MOV34 (JAMM) domain metalloproteases (metalloprotease DUMBs) are highly specific for K63 poly-Ub linkage [71] and regulate proteolysis via directly interacting with E3 ligase, modulating the level of substrate ubiquitination, hydrolyzing/remodeling ubiquitinated substrates, altering target protein localization, and acting on proteasome-bound substrates [70,72,73,74]. Thus, the downregulation of the metalloprotease DUBs pathway in MMC-treated HCAECs leads to the disruption of post-translational protein modification, the failure of cellular signaling and other processes that ensure the normal functioning of endothelial cells, and, finally endothelial dysfunction.

In our experiment we identified four pathways that were downregulated in HCAECs in response to genotoxic stress and were involved in membrane trafficking—a part of the vesicle-mediated transport pathway. Clathrin-mediated endocytosis (CME) controls the uptake of molecules (metabolites, hormones and other proteins) from the extracellular space and plasma membrane with the formation of clathrin-coated vesicles, regulates membrane composition by recycling membrane components and/or targeting them for degradation [75,76] and takes part in signal transduction by regulating the expression and signaling of receptor tyrosine kinases (RTKs) and G-protein coupled receptors (GPCRs) on the cell surface [77,78]. The recruitment of molecules localized on the plasma membrane (called “cargo”) into clathrin-coated endocytic vesicles is mediated by interactions with a variety of clathrin-associated sorting proteins (CLASPs) that bridge the recruitment of cargo to clathrin-coated vesicles [79]. We can suppose that the downregulation of the vesicle-mediated transport pathway in MMC-treated HCAECs leads to the impaired intracellular transport of molecules and cellular signaling followed by the failure of endothelial homeostasis.

Interestingly, HCAECs and HITAECs are characterized by different molecular responses to MMC-induced genotoxic stress. Here, we found that MMC-treated HITAECs are characterized by the downregulation of the mitochondrial ABC (ATP-binding cassette) transporters localizing in mitochondria and playing a role in preventing oxidative stress [80,81]. In summary, the downregulation of this pathway may lead to an impaired energy metabolism and apoptosis [82].

Transcription regulation is one of the pathways involved in the response of HITAECs to MMC-induced genotoxic stress. Small nuclear RNA (snRNA) plays key roles in splicing, as the U1, U2, U4, U4atac, U5, U11, and U12 snRNAs are transcribed by RNA polymerase II [83]. It has been shown that snRNA can regulate the expression of endothelial nitric oxide synthase (eNOS) [84]—one of the key markers of endothelial dysfunction. Non-coding RNAs (including snRNAs) also control cell proliferation, the degradation of the extracellular environment, and the endothelial-to-mesenchymal transition (another significant marker of endothelial dysfunction) [85]. Another way to regulate gene expression and other cellular process involves nuclear receptors—ligand-activated transcription factors that bind to small lipid-based molecules [86]. Thus, it can be suggested that one of the main mechanisms underlying MMC-induced endothelial dysfunction in HITAECs is an impaired transcription due to the downregulation of the corresponding pathways.

It is known that the human coronary artery is most often affected by atherosclerosis, while atherosclerotic lesions of the internal thoracic artery are quite rare [87] due to their hydrodynamic features [88] and the molecular heterogeneity of these vessels [89]. We can suggest that molecular heterogeneity is mainly responsible for the differences in the molecular responses of HCAECs and HITAECs to MMC-induced genotoxic stress. As concluded from the results of the present study, HITAECs are more resistant to genotoxic stress compared to HCAECs, which was manifested in the smaller number of DEPs and upregulated/downregulated molecular pathways identified in this cell line. In other words, genotoxic stress in HITAECs leads to fewer changes in their molecular pattern, less impairment of endothelial homeostasis, and, finally, a decreased risk of atherosclerotic lesions in the internal thoracic arteria.

## 4. Materials and Methods

### 4.1. Cell Culture

Commercially available HCAECs (cat. No. 300K-05a, Cell Applications Inc., San Diego, CA, USA) and HITAECs (cat. No. 308K-05a, Cell Applications Inc., San Diego, CA, USA) cryopreserved at the 2nd passage were used in the present research. All manipulations with cells were performed as described previously [27]. Briefly, the cells were seeded into fibronectin-coated T-75 flasks (Greiner Bio-One GmbH., Kremsmünster, Austria) containing 15 mL of a Human MesoEndo Cell Growth Medium (Cell Applications Inc., San Diego, CA, USA) and incubated in a humidified atmosphere with 5% CO_2_ at 37 °C. After 3 passages, the cells were reseeded into new T-75 flasks (Greiner Bio-One GmbH., Kremsmünster, Austria) and refed (after reaching 80% confluency) with another 15 mL of a Human MesoEndo Cell Growth Medium (Cell Applications Inc., San Diego, CA, USA) containing 500 ng/mL of MMC (AppliChem, Barcelona, Spain, CAS No. 50-07-7) (treatment group) or 0.9% NaCl (control group) followed by 6 h of incubation. Then, cells were washed twice using ice-cold phosphate-buffered saline (PBS) and refed with another 15 mL of an additive-free Human MesoEndo Cell Growth Medium (Cell Applications Inc., San Diego, CA, USA) followed by 24 h of incubation. To avoid any possible batch-effects, all manipulations with HCAECs and HITAECs were performed in parallel.

### 4.2. Protein Isolation

To perform protein isolation, a culture medium was removed from culture flasks; the cells (approximately 3 million cells per one culture flask) were washed twice using ice-cold PBS and lysed with 500 μL of a radioimmunoprecipitation assay (RIPA) buffer (Thermo Fisher Scientific, Waltham, MA, USA) supplied with a Halt protease and phosphatase inhibitor cocktail (ThermoFisher Scientific, Waltham, MA, USA) in accordance with the manufacturer’s protocol. The obtained cell lysate was centrifuged using a Microfuge 20R centrifuge (Beckman Coulter, Brea, CA, USA) for 15 min at 14,000× *g* and 4 °C; the supernatant was transferred into a clean 1.5 mL Eppendorf tube. The quantity of the isolated protein was evaluated via a BCA Protein Assay Kit (Thermo Fisher Scientific, Waltham, MA, USA) using a Multiskan Sky microplate spectrophotometer (ThermoFisher Scientific, Waltham, MA, USA) according to the manufacturer’s protocol.

### 4.3. Sample Preparation for Proteomic Profiling

To perform tryptic digestion, an RIPA buffer was removed using 1 h of acetone (Sigma-Aldrich, Saint Louis, MO, USA) precipitation at −20 °C followed by the centrifugation of samples for 15 min at 13,000× *g* and 4 °C. The supernatant was aspirated and the protein pellet was resuspended in 250 μL of acetone (Sigma-Aldrich, Saint Louis, MO, USA) for 15 min at −20 °C followed by centrifugation for 15 min at 13,000× *g* and 4 °C; the supernatant was removed and the pellet was air dried for 5–10 min. Next, the pellet was resuspended in 8 mol/L of urea (Sigma-Aldrich, Saint Louis, MO, USA) and 50 mmol/L of ammonium bicarbonate (Sigma-Aldrich, Saint Louis, MO, USA), incubated for 20 min at 4 °C, ultrasonicated in a water bath and incubated for another 10 min at 4 °C. The protein was quantified by a Qubit 4 fluorometer (ThermoFisher Scientific, Waltham, MA, USA) using a QuDye Protein Quantification Kit (Lumiprobe, Cockeysville, MD, USA) in accordance with the manufacturer’s protocol. A total of 15 μg of protein were dissolved in 5 mmol/L of dithiothreitol (Sigma-Aldrich, Saint Louis, MO, USA) and incubated for 1 h at 37 °C followed by another 30 min of incubation with 15 mmol/L of iodoacetamide (Sigma-Aldrich, Saint Louis, MO, USA) in the dark at room temperature. Next, the samples were diluted with 7 volumes of 50 mmol/L of ammonium bicarbonate (ThermoFisher Scientific, Waltham, MA, USA) supplied by 300 ng of trypsin (Promega, Madison, WI, USA) and incubated for 16 h at 37 °C. The desalting of the samples was performed using 200 μL stagetips with an RPS sorbent (Affinisep, Le Houlme, France) using methanol (Sigma-Aldrich, Saint Louis, MO, USA), acetonitrile (Sigma-Aldrich, Saint Louis, MO, USA) and 0.1% formic acid (Sigma-Aldrich, Saint Louis, MO, USA) in accordance with the manufacturer’s protocol. Finally, the samples were dried using a centrifuge concentrator (Concentrator plus, Eppendorf, Hamburg, Germany) for 3 h and dissolved in water for a chromatography (Sigma-Aldrich, Saint Louis, MO, USA) supplied with 0.1% formic acid (Sigma-Aldrich, Saint Louis, MO, USA).

### 4.4. Proteomic Profiling

Proteomic profiling was performed by UHPLC-MS/MS with ion mobility in the Centre for Molecular and Cell Technologies (Saint Petersburg State University, Saint Petersburg, Russia). In total, four biological replicates were analyzed in each studied group. Approximately 500 ng of peptides per sample were used for shotgun proteomics analysis in a TimsToF Pro mass spectrometer with a nanoElute UHPLC system (Bruker, Billerica, MA, USA). UHPLC was performed in the one-column separation mode with an Aurora Ultimate 25 cm separation column (C18 stationary phase, 250 mm × 0.075 mm, 1.7 μm, 120 A; IonOpticks, Fitzroy, Australia) in a gradient mode with a 300 nL/min flow rate and with the column temperature set at 60 °C. Phase A was water/0.1% formic acid (Sigma-Aldrich, Saint Louis, MO, USA) and phase B was acetonitrile/0.1% formic acid (Sigma-Aldrich, Saint Louis, MO, USA). The following gradient was used: (i) from 2% to 18% phase B for 44 min, (ii) to 25% of phase B for 11 min, (iii) to 37% of phase B for 5 min and then (iv) to 85% of phase B for 2 min followed by washing with 85% phase B for 15 min. Before each sample, trap and separation columns were equilibrated with 4 column volumes. Next, electrospray ionization with the 0.4 bar pressure of the nebulizer, 4500 V of capillary voltage, 3 l/min N2 flow, and an 180 °C ion source temperature was performed. The mass spectrometry acquisition was performed in automatic DDA PASEF (data-dependent acquisition parallel accumulation serial fragmentation) mode with a 1.1 s cycle in positive polarity with the fragmentation of ions with at least two charges in an m/z range from 100 to 1700 and an ion mobility range from 0.60 to 1.60 1/K0.

### 4.5. Protein Identification

Protein identification was performed using Peaks Xpro v.10.6 software (Bioinformatics Solutions Inc., Waterloo, ON, Canada). Proteins characterized by a parent mass error tolerance of 10 ppm and a fragment mass error tolerance of 0.05 ppm, an FDR < 1%, two possible missed cleavage sites, and the presence of at least two unique peptides were selected for further analysis. Cysteine carbamidomethylation was set as fixed modification; methionine oxidation, N-terminal acetylation, asparagine and glutamine deamidation were set as variable modifications. For data analysis, we used the human protein SwissProt database (uploaded on 20 January 2024; accessed on 1 March 2024) and the cRAP contaminants database (version of 4 March 2019; accessed on 1 March 2024).

### 4.6. Statistical Analysis

Peak areas were used for quantitative data analysis. Statistical analysis of the data was performed in R v.4.1.2 [90]. Proteins with more than 2/3 missing values were excluded from the analysis. To eliminate the missing values from other samples, the k-nearest neighbors (kNN) algorithm [91] was performed using the «impute» package. Then, log2 transformation and quantile normalization [92] were applied. Differential expression analysis was performed using the «limma» package (v.3.50.3) [93]. Principal component analysis (PCA) was performed using the «mixOmics» package (v.6.18.1) [94]. The «ggplot2» (v.3.4.4) [95] and «EnhancedVolcano» (v.1.12.0) [96] packages were used for data visualization. Pathway enrichment analysis of DEPs was performed using the Gene Ontology (https://geneontology.org/, accessed on 6 March 2024) and Reactome (https://reactome.org/, accessed on 6 March 2024) databases with a relevant reference list for *Homo sapiens* that was current as of 6 May 2024.

## 5. Conclusions

The upregulation of the processes of nucleotides and RNA metabolism, and the downregulation of the processes of vesicle-mediated transport, post-translation protein modification, cell cycle control, the transport of small molecules, transcription and signal transduction are the most significant pathways underlying genotoxic stress-induced endothelial disfunction in in vitro models. The obtained results could improve our understanding of the fundamental basis of atherogenesis and help in the justification of genotoxic stress as a risk factor for atherosclerosis. The deciphering of the molecular pathways underlying genotoxic stress-induced endothelial dysfunction makes it possible to correct existing atherosclerosis therapies, as well as to develop new treatment strategies based on targeted actions on the molecular pathways involved in endothelial dysfunction mainly in populations in regions with high genotoxic backgrounds.

## Figures and Tables

**Table 1:** Pathways enriched in HCAECs upon MMC treatment (according to the Reactome Pathways Database, accessed on 6 March 2024).

Pathway Name (Reactome Identifier)	Total Number of Proteins	Number of Denoted Proteins	Percent from DEPs	FDR-Corrected *p*-Value
**Upregulated after MMC treatment**				
Phosphate bond hydrolysis by NUDT proteins (R-HSA-2393930)	11	4	4.3	6.28 × 10^−4^
Purine catabolism (R-HSA-74259)	28	4	4.3	0.012
Tristetraprolin (TTP, ZFP36) binds and destabilizes mRNA (R-HSA-450513)	258	8	8.5	0.041
Nucleotide catabolism (R-HSA-8956319)	46	4	4.3	0.041
**Downregulated after MMC treatment**				
Sealing of the nuclear envelope (NE) by ESCRT-III (R-HSA-9668328)	94	7	6.5	0.003
Cargo recognition for clathrin-mediated endocytosis (R-HSA-8856825)	157	8	7.4	0.004
Clathrin-mediated endocytosis (R-HAS-8856828)	213	9	8.3	0.004
Metalloprotease DUBs (R-HSA-5689901)	103	6	5.6	0.017
Lysosome Vesicle Biogenesis (R-HSA-432720)	794	15	13.9	0.044
Trans-Golgi Network Vesicle Budding (R-HSA-199992)	987	17	15.7	0.044

**Note:** HCAECs, Human Coronary Artery Endothelial Cells; DEPs, differentially expressed proteins; FDR, false discovery rate; MMC, mitomycin C.

**Table 2:** Pathways enriched in HITAECs upon MMC treatment (according to the Reactome Pathways Database, accessed on 6 March 2024).

Pathway Name	Total Number of Proteins	Number of Denoted Proteins	Percent from DEPs	FDR-Corrected *p*-Value
**Downregulated after MMC treatment**				
Mitochondrial ABC transporters (R-HSA-1369007)	4	2	10.0	0.009
RNA polymerase II transcribes snRNA genes (R-HSA-6807505)	220	4	20.0	0.033
NR1H2 & NR1H3 regulate gene expression linked to lipogenesis (R-HSA-9029558)	84	3	15.5	0.033

**Note:** HITAECs, Human Internal Thoracic Artery Endothelial Cells; DEPs, differentially expressed proteins; FDR, false discovery rate; MMC, mitomycin C.

**Table 3:** Distribution of DEPs identified in MMC-treated HCAECs between functional groups according to GO enrichment analysis.

Molecular Term	Number of Denoted Proteins	Percent from DEPs	Fold Enrichment	FDR-Corrected *p*-Value
**Upregulated after MMC treatment**				
Response to platinum ion (BP)	2	2.13	>100	4.95 × 10^−2^
Nucleic acid metabolic process (BP)	24	25.53	2.41	3.06 × 10^−2^
Organic substance biosynthetic process (BP)	36	38.30	1.99	2.37 × 10^−2^
Cellular component organization (BP)	45	47.87	1.75	2.64 × 10^−2^
RNA binding (MF)	22	23.40	2.87	1.27 × 10^−2^
Protein binding (MF)	86	91.49	1.30	5.75 × 10^−3^
Organelle envelope (CC)	17	18.09	2.88	9.80 × 10^−3^
Nucleoplasm (CC)	38	40.43	2.00	2.33 × 10^−3^
Cytosol (CC)	47	50.00	1.86	9.30 × 10^−4^
Protein-containing complex (CC)	48	51.06	1.61	1.59 × 10^−2^
**Downregulated after MMC treatment**				
Multivesicular body assembly (BP)	5	4.63	30.64	5.17 × 10^−3^
Protein metabolic process (BP)	4	3.70	2.32	2.80 × 10^−4^
Cellular biosynthetic process (BP)	42	38.89	2.13	4.02 × 10^−3^
Postsynaptic endocytic zone cytoplasmic component (CC)	2	1.85	>100	4.02 × 10^−2^
ESCRT III complex (CC)	3	2.78	53.49	3.22 × 10^−2^
Amphisome membrane (CC)	3	2.78	49.03	4.28 × 10^−2^
Multivesicular body membrane (CC)	4	3.70	27.05	2.15 × 10^−2^
Clathrin vesicle coat (CC)	4	3.70	22.41	4.62 × 10^−2^

**Note:** HCAECs, Human Coronary Artery Endothelial Cells; DEPs, differentially expressed proteins; GO, Gene Ontology; FDR, false discovery rate; MMC, mitomycin C; BPs, Biological Processes; MFs, Molecular Functions; CCs, Cellular Components.

## Data Availability

The mass spectrometry proteomics data have been deposited to the ProteomeXchange Consortium via the PRIDE partner repository with the dataset identifier PXD050743 (project DOI: 10.6019/PXD050743).
